# A Review of Data Gathering Methods for Evaluating Socially Assistive Systems

**DOI:** 10.3390/s22010082

**Published:** 2021-12-23

**Authors:** Shi Qiu, Pengcheng An, Kai Kang, Jun Hu, Ting Han, Matthias Rauterberg

**Affiliations:** 1 Department of Design, Shanghai Jiao Tong University, Shanghai 200240, China; hanting@sjtu.edu.cn; 2 Department of Industrial Design, Eindhoven University of Technology, 5600MB Eindhoven, The Netherlands; k.kang@tue.nl (K.K.); J.Hu@tue.nl (J.H.); G.W.M.Rauterberg@tue.nl (M.R.); 3 School of Design, Southern University of Science and Technology, Shenzhen 518055, China; pengcheng.an@uwaterloo.ca; 4 School of Computer Science, University of Waterloo, Waterloo, ON N2L 3G1, Canada; 5 Department of Industrial Design, Nantong University, Nantong 226019, China

**Keywords:** assistive technology, social interaction, data gathering methods, older adults, people with disabilities

## Abstract

Social interactions significantly impact the quality of life for people with special needs (e.g., older adults with dementia and children with autism). They may suffer loneliness and social isolation more often than people without disabilities. There is a growing demand for technologies to satisfy the social needs of such user groups. However, evaluating these systems can be challenging due to the extra difficulty of gathering data from people with special needs (e.g., communication barriers involving older adults with dementia and children with autism). Thus, in this systematic review, we focus on studying data gathering methods for evaluating socially assistive systems (SAS). Six academic databases (i.e., Scopus, Web of Science, ACM, Science Direct, PubMed, and IEEE Xplore) were searched, covering articles published from January 2000 to July 2021. A total of 65 articles met the inclusion criteria for this systematic review. The results showed that existing SASs most often targeted people with visual impairments, older adults, and children with autism. For instance, a common type of SASs aimed to help blind people perceive social signals (e.g., facial expressions). SASs were most commonly assessed with interviews, questionnaires, and observation data. Around half of the interview studies only involved target users, while the other half also included secondary users or stakeholders. Questionnaires were mostly used with older adults and people with visual impairments to measure their social interaction, emotional state, and system usability. A great majority of observational studies were carried out with users in special age groups, especially older adults and children with autism. We thereby contribute an overview of how different data gathering methods were used with various target users of SASs. Relevant insights are extracted to inform future development and research.

## 1. Introduction

People are inherently social since they live, play, work together, and socialize [1]. Social interactions significantly influence the quality of life of people in general and people with special needs in particular [2]. Over the last two decades, many empirical studies in public health have shown that the quantity of social interactions is positively correlated with personal health [3]. Social interactions can benefit personal health from several aspects, such as the transmission of health information and promoting healthy behaviors [3]. Otherwise, the lack of sufficient social interactions in daily routines can have a negative impact on health, such as mental health problems caused by social isolation [4]. Many examples can be seen regarding social problems people face with special needs. Limited or poor social interactions have been found to increase the risk of dementia by 60 percent [5]. Recently, loneliness, social isolation, and even poor health have become more severe with the COVID-19 pandemic. Older adults at high risk are asked to “stay home as much as possible” [6] and to follow social distancing measures to prevent infection. Social problems can also be found regarding blind people. As reported in [7], because blind people are limited in the social cues they can send and receive in face-to-face conversations, they tend to be less confident than sighted people with respect to communicating their feelings. They appeared to be introverted, submissive, and with low confidence in social situations. Additionally, children with autism spectrum disorder (ASD) lack sufficient social ability to respond correctly to social conversations, understand the feelings of conversation partners, and react appropriately in a social situation [8,9,10,11,12]. Social interaction problems often cause children with ASD to experience difficulties in their day-to-day lives [12].

Social isolation and loneliness have negative impacts on people with special needs, such as causing poor health, disability, and even death. Unfortunately, many healthcare systems cannot meet their needs regarding supporting social interactions. Thus, there is substantial demand for developing assistive technologies that support their social needs. According to the World Health Organization (WHO), the primary purpose of assistive technology is to “maintain or improve an individual’s functioning and independence to facilitate participation and to enhance overall well-being” [13]. Assistive technologies include many technologies and products, from simple devices to complicated high-tech systems [14]. Additionally, the International Organization for Standardization (ISO) defines an assistive product as “any product (including devices, equipment, instruments, and software) … especially produced or generally available, used by or for persons with disabilities” [15], which considers broadening the target user groups. In this review, assistive technology refers to the information system, which is supposed to support and be used directly by people with functional limitations, typically older adults and people with disabilities. 

With the development of assistive technology, there is a growing number of socially assistive systems (SAS) to aid people with special needs in developing positive social interactions with others [16]. The emergence of a diversity of social and communication technologies has changed how people live, i.e., how they keep in touch and establish their social network [1]. Such technologies enable conventional face-to-face conversations to be different kinds of computer-based conversations at a distance, and new user interface forms facilitate face-to-face social interactions [1]. For example, in [17], the researchers developed an online platform to increase the intergenerational interactions between distant grandparents and their grandchildren. The social support aid (SSA) helped people with memory loss remember the names of people and relationships that they interacted within social activities, and therefore helped to enhance social engagement [18]. An enhanced social awareness (ESA) network has been used to aid blind people in identifying friends and initiating social contact. In [19], a “social glasses” prototype system helped blind people perceive a gaze from a sighted conversation partner and react to her a simulated gaze, to promote communication quality. As reported in [20], a facial expression recognition system was implemented based on google glasses to provide social cues for children with ASD. 

There is growing interest in developing SAS, and every year many new studies are published. As we have not identified systematic reviews that focused on assistive technologies for support social interactions other than robotic systems ([21,22,23,24]), there is a need to categorize the current research studies. Hence, we have outlined the research methods used to study SASs, identify the outcomes, inform design practice, and guide further research. In a prior work [25], we mainly reported using human-computer interaction (HCI) technologies for SASs and the general categories of system evaluation. In this review, we mainly focus on data gathering methods for assessing SASs. In HCI, data gathering is considered to be essential for system evaluation, capturing users’ reactions and performances with a system or prototype [1]. According to [1], interviews, questionnaires, and observation are three basic techniques for gathering data in HCI: (a) interviews involve an interviewer asking users a set of structured or unstructured questions, (b) questionnaires consist of a series of questions for users to answer, (c) observations involves observing users’ activities as they happens or makes a record to be studied later. Aside from these three major techniques, other methods include user task performance, system log, physiological data, etc. Generally, such basic data gathering methods can be used in combination with evaluating one particular SAS to avoid biases in any single method [1]. 

A fundamental challenge associated with investigating SASs concerns evaluating the developed technology. Researchers often have limited access to users with special needs, and extra efforts and care are usually required to gather data from these users. Therefore, a systematic review on how existing studies evaluated SASs with various user groups could inform future researchers and developers of the methods and challenges in current practice and support them in planning their evaluation activities in research. However, few previous literature reviews on assistive technologies have focused explicitly on this relevant yet challenging aspect. In this review, we aim to offer a comprehensive overview of the following: (a) how a broad range of SASs have been evaluated with their target user groups (or stakeholders) and (b) what methods have been used to gather data from various user groups with special needs. Firstly, we should know the target users that SASs are mainly focused on as the premise. Secondly, we investigate how different data gathering methods are used with various target users of SASs.

In summary, we primarily investigate the following research questions: 

**RQ1**: Who are the target users that SASs are mainly focused on?

**RQ2**: How different data gathering methods are used with various target users to evaluate SASs?

## 2. Method

### 2.1. Data Collection

We used the same search strategy as described in detail in [25]. In 2019, a literature search was conducted to explore the data gathering methods of SASs. Due to the updates of the databases, the second literature search was performed in July 2021. Finally, the time covered in this search was January 2000 to July 2021. 

#### 2.1.1. Search Terms

**Figure 1:**
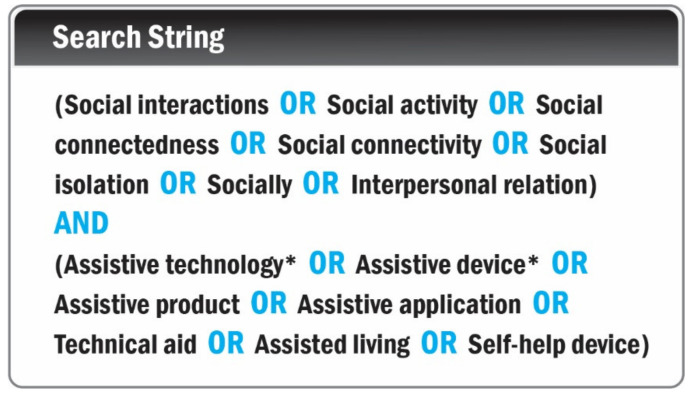
Boolean search string used in the search strategy.

According to the previous study [25], we used two categories of the medical subject heading (mesh) terms: “social interactions” and “assistive technologies”. Synonyms and spelling variations of these two MeSh terms were used in several combinations and modified for the six databases. Figure 1 shows the search strategy.

#### 2.1.2. Databases Searched

The relevant articles were searched from the following six databases that are often used by researchers in the HCI community: Scopus, Web of Science, ACM, Science Direct, PubMed, and IEEE Xplore. These databases provide full-text journals and the most important conference publications, including assistive technologies, social interactions, and their relations. 

### 2.2. Article Selection

The procedure of article selection followed the guidelines of The Preferred Reporting Items for Systematic Reviews and Meta-Analyses (PRISMA) Statement [26] as follows:

- A computerized search strategy (Figure 1) was conducted for the period from October 2019 to July 2021.
- S.Q. removed duplicates and evaluated titles and abstracts against the inclusion and exclusion criteria.
- According to our inclusion and exclusion criteria, two independent coders (S.Q. and P.C.A.) conducted the full-text review. Finally, discrepancies were resolved by discussions between two coders.

Inclusion and Exclusion Criteria are presented as follows:

**Inclusion Criteria** Studies were included if they were empirical studies that focused on system design or user evaluation of SASs.

**Exclusion Criteria** Studies were excluded if they (a) were reviews, theoretical articles, concept articles, or market survey; (b) were not written in English or less than four pages; (c) were duplicate reports of the same study in different sources; (d) reported no information system; (e) reported assistive information systems, but not for social interactions; (f) studied target users who were not older adults and people with disabilities; (g) reported robotic systems. Since several existing systematic review studies have reported socially assistive robots for older adults [21,22,23] and children [24], we excluded robotic systems in this review. 

### 2.3. Data Analysis

Studies were coded in terms of (a) name of authors and date published, (b) research purpose, (c) target users, and (d) methods of data gathering. The template including the above categories in more detail is shown in Appendix A (Table A1). In this review, we focus on analyzing target users and data gathering methods as follows:

**Target users** Typically include older adults and people with different types of impairments, disabilities, and handicaps.

**Data gathering** includes the following categories:

(1) Interview data are regarding user experience, expert opinions, or stakeholder opinions.

(2) Questionnaires consist of standardized questionnaires and self-developed questionnaires.

(3) Observational data is objective and should avoid preconceived notions, generally collected from taking notes, photos, and making video or audio recordings of the sessions [27].

(4) User task performance refers to users’ cognitive tasks and bodily tasks, such as accuracy and score.

(5) System log describes the usage of the system, such as time and frequency of the system used.

(6) Functional validation is primarily about system recognition rates.

(7) Physiological data consist of heart rate variability (HRV), body movements, gaze data, etc.

## 3. Results

### 3.1. Overview

A total of 1463 articles were identified according to the keywords searched from the six databases (i.e., Scopus, Web of Science, ACM, Science Direct, PubMed, and IEEE Xplore). A computerized search strategy was conducted from October 2019 to July 2021. The first literature search was performed in October 2019 and lasted around one and half a month. Due to the updates of the six databases, we completed the second literature search in July 2021, which lasted around one month. 

In the beginning, because of searching articles from the six databases, two or several databases might find the same qualified article. The duplicated articles should be removed. Removing duplicated articles did not require the application of any inclusion or exclusion criteria. After removing duplicated records, 1143 articles were considered for the title and abstract screening.

In the first round of screening, S.Q. read the title and abstract of 1463 articles and screened them by applying the inclusion and exclusion criteria. After that, 978 articles were excluded after reading the title and abstract, and 165 articles remained. In the second round of screening, still applying the same inclusion and exclusion criteria, two independent coders (S.Q. and P.C.A.) read the full-text of 165 articles, and completed an assessment. Thus, we included 56 of 165 articles. Additionally, the researchers manually searched the references for the included articles, and nine articles were identified after hand searching references. Finally, 65 articles were considered suitable for analysis. 

**Figure 2:**
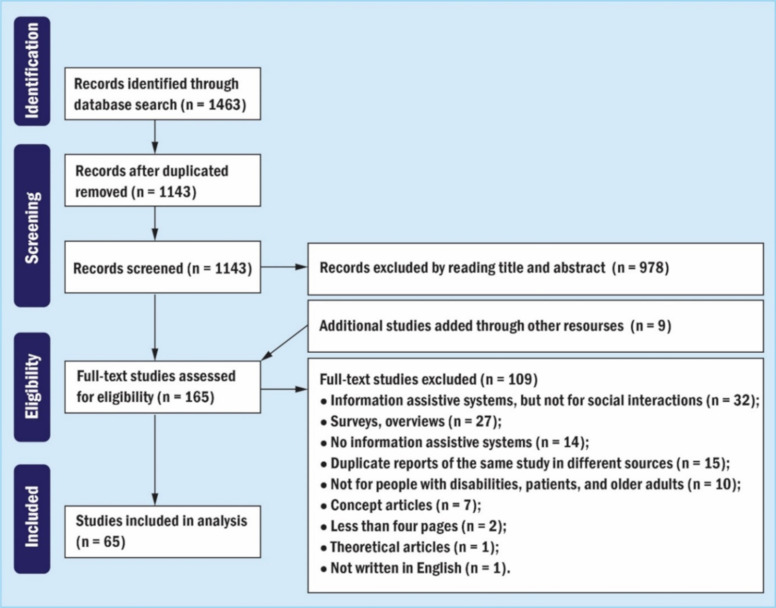
PRISMA flow chart of our dataset according to [26].

In the first and second rounds of screening, the same inclusion and exclusion criteria were used. The critical difference was that the articles were selected based on their title and abstract in the first round. The articles were selected based on their full-text in the second round. Figure 2 shows the overall study selection procedure and the number of articles excluded or included in each step. 

**Figure 3:**
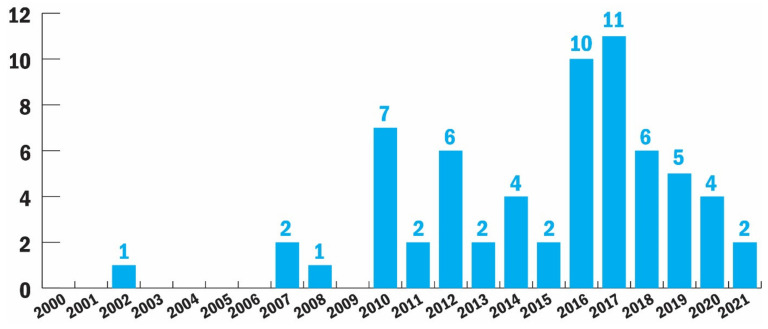
The number of articles according to the year of publication.

Figure 3 shows the number of articles on SASs from January 2000 to July 2021. Before 2010, only four articles were published that met our selection criteria. After 2010, 61 out of 65 articles were published in the last 12 years. This increase suggests that SASs have gained increasing attention in the HCI community in the recent 12 years. Appendix A shows all the studies included in detail.

### 3.2. Target Users

As shown in Table 1, we inventoried the target population addressed by SASs. Appendix B illustrates the published journals and conference papers during 2000–2021 regarding SASs according to the classification of the target population. Two major categories in Table 1 can be identified: older adults (21 studies) and people with disabilities (44 studies). The category “older adults” included two subcategories: (a) older adults in general and (b) older adults with cognitive impairments. Most studies (14/21) reported SASs for older adults in general, while only seven studies investigated older adults with dementia (N = 6 studies) and mild cognitive impairment (N = 1 study).

The category “people with disabilities” consisted of six subcategories: (a) visual impairments (20 studies), (b) ASD (9 studies), (c) listening and speech impairments (7 studies), (d) mobility and physical impairments (5 studies), as well as (e) others (3 studies). In more detail, among 20 studies of people with visual impairments, the most common (14/20) is to develop SASs to help blind people perceive social signals, including identifying facial expressions of interaction partners [28,29,30,31,32], or sensing eye gaze from sighted people [33,34]. Additionally, in [19], the researchers presented the idea of artificial eyes, to establish “eye contact” between a blind person and a sighted conversation partner. Nine studies developed SASs for children with ASD [20,35,36,37,38,39,40,41,42], indicating an early intervention in autism. Among seven studies of people with listening and speech impairments, most of them (5/7) developed SASs for people with deafness [43,44,45,46,47]. The other two studies focused on deaf-and-dumb people [48] and people with speech or language impairment [49]. Five studies reported SASs for people with mobility and physical impairments, including physical disability [50,51,52], motion disability [53], and spinal cord injury [54]. Other studies included target users with severe speech and physical impairments (SSPI) [55], social communication disorders (SCD) [56], as well as low vision, Alzheimer’s disease, and ASD [57].

### 3.3. Data Gathering

**Figure 4:**
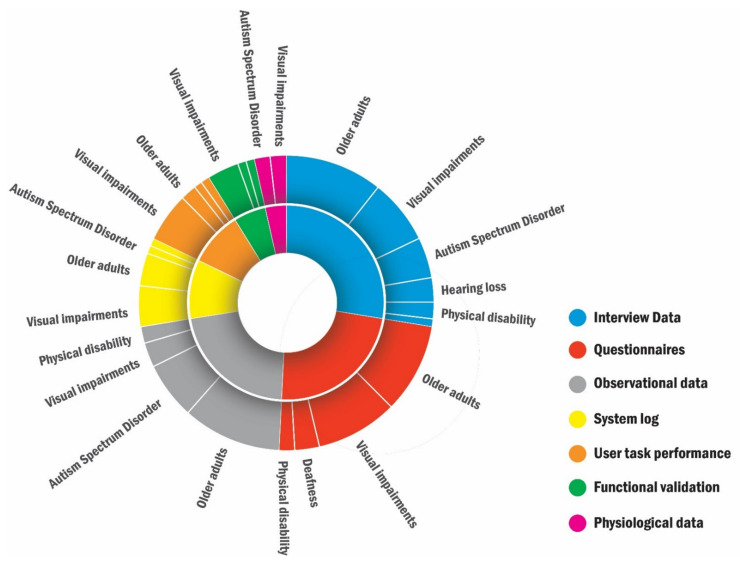
Data gathering methods for target users.

Data gathering included seven types (Table 2): interview data (31 studies), questionnaires (26 studies), observational data (24 studies), system log (11 studies), user task performance (10 studies), functional validation (6 studies), and physiological data (4 studies). Figure 4 shows the data gathering methods for primary target users. 

#### 3.3.1. Interview Data

A total of 31 studies gathered interview data; 27 of these studies organized (one-on-one) personal interviews, while seven studies [34,45,59,60,68,70,72] conducted group interviews (e.g., group discussions or focus groups); among them, three studies, i.e., [59,60,68], were organized to complement both personal and group perspectives. Context wise, the primary target group of these studies consisted of older adults (12 studies with 4 studies focused on older adults with dementia), children with ASD (N = 5 studies), people with visual impairment (N = 8 studies), people with hearing loss (N = 3 studies), physical disability (N = 2 studies), or communication disorder (N = 1 study). Interestingly, while 16 studies focused on gathering interview data mainly from primary user groups, the other 15 studies also gathered interview data from secondary users, alternative users, or relevant stakeholders from the target context. For instance, in seven (out of twelve) studies targeted at older adults, i.e., [18,59,60,63,64,70,71], the perspectives of caregivers, friends, and family members were also gathered. Regarding the data gathered in [71], the opinions of activity facilitators of older adults with dementia were heavily relied on. Similarly, two studies on children with ASD also gathered perspectives from the children’s mothers, in [35], and school teachers, staff members, and autism specialists, in [40]. As for studies aimed at assisting social communication of people with visual or hearing impairment, the experience and perception of the primary users’ social counterparts were sometimes the objectives of the data gathering. Namely, studies by [33,78,80] gathered experiences of sighted people who interacted with the visually impaired group, while [43] explored the perception of conversation partners about the assistive system designed for people with hearing loss.

The semi-structured interview seemed to be the most common approach applied by the 27 studies with personal interviews: (a) 10 studies, i.e., [18,35,39,44,60,64,71,76,80,84], explicitly mentioned that they had organized semi-structured interviews, whereas (b) the majority of the other studies also seemed to follow a semi-structured manner according to their description of the interview questions (which often exhibited an open-ended, explorative nature). However, most studies only had a passing description of the interview process. A few studies had limited, or rather weak, descriptions on how the interviews had been conducted, i.e., [45,53,56,59,70,78,87]. 

By contrast, four studies, i.e., [44,60,83,84] provided relatively detailed descriptions of the protocol or process of the conducted interviews. In addition, the majority of the studies in this section employed a face-to-face setting for the conducted personal or group discussions, whereas three studies, i.e., [53,59,84], involved remote or online interviews. For instance, in [84], researchers carried out interviews with participants living with visual impairments over Skype or telephone. Participants from a broad range of geographical locations could be reached across the USA and Canada. For another example, in [53], a remote conferencing assessment was conducted with an extra user with (motion) disability.

The interview data gathered by all the studies were reported to be transcribed verbatim for qualitative analyses. However, only a small portion of the studies (11 out of 30) further characterized the type of the applied analysis method instead of referring to the generic term “qualitative analysis”. The most reported analysis methods were thematic analysis [90] and grounded theory [91]. Namely, four studies, i.e., [18,54,64,72], applied thematic analyses, with two studies, i.e., [18,64], citing the standard steps formulated by Braun and Clarke [90]. Another five studies, i.e., [38,39,43,62,84], opted for a grounded theory method, referring to the original procedure introduced by Chamaz [92] or Strauss and Corbin [91]. In addition, a few other qualitative analysis methods were also mentioned, for example, affinity analysis [93] (applied in [39]), content analysis (by [90,94]) (used in [68]), as well as constant comparative analysis [90] (applied in [72,76]). A number of studies provided little information concerning the processes of their qualitative analysis, i.e., [44,56,59,78,83,87]. Only a few studies (i.e., [38]) addressed the data analysis process at a relatively detailed level. Seven studies, i.e., [18,39,43,60,64,76,84], explicitly mentioned the involvement of more than one analyst (or coder) in the analysis process. For example, [39] employed two coders to independently process the whole dataset. [78] used an extra facilitator to compile the coding framework, while the lead analyst mainly carried out the coding, and in the studies of [39,43,84], the coders collaborated to share the work separately or in joint coding sessions and finalized the results by cross-checking and group discussion. Studies by [60,76] did not seem to have an explicit coding phase but relied on a joint summarization or discussion of the qualitative data by the research team.

#### 3.3.2. Questionnaires

A total of 26 studies used questionnaires to collect data. These studies were targeted at older adult, i.e., [17,18,58,60,61,62,63,64,65,66,69], people with visual impairments, i.e., [19,28,29,33,34,77,80,83,85,87], deafness, i.e., [43,46,48], and physical disabilities, i.e., [50,54]. A total of 34 questionnaires (Table 3) from 26 studies consisted of two categories: (a) standardized questionnaires (N = 21 questionnaires) and (b) self-developed questionnaires (N = 13 questionnaires). These questionnaires measure users’ perceptions of using SASs from two aspects: (a) a social interaction and emotional state and (b) a usability-related aspect. 

(I) Standardized questionnaires

Among all the studies, we found 11 standardized questionnaires for measuring social interactions. These questionnaires include Inclusion of Other in the Self (IOS) scale, in [19,34,62,64]; Affective Benefits in Communication (ABC) in [63], User Engagement Scale (UES) in [17], Lubben Social Network Scale (LSNS) in [65], Two-Dimensional Social Interaction Scale (2DSIS) in [80], Networked Minds Social Presence Inventory (NMSPI) in [19], Psychosocial Impact of Assistive devices Scale (PIADS) in [54], Life Habits Assessment (Life-H) in [46], Functional Assessment of Communication Skills for Adults (FACS-A) in [46], Interpersonal Attraction Scales (IAS) in [61], and Intrinsic Motivation Inventory questionnaire (IMI) in [34]. Among them, the IOS was used by multiple studies, i.e., [19,34,62,64], for measuring closeness. The IOS is a seven-point pictorial scale using two overlapping circles. More overlapping circles indicate higher levels of relationship closeness between two people. Other standardized questionnaires assess various aspects of social interactions, such as social engagement (LSNS), social presence (NMSPI), and interpersonal attraction (IAS).

Seven standardized questionnaires were used for measuring the emotional state of the participants, such as loneliness in [65], depression in [65,66], as well as positive and negative effects in [66]. These questionnaires are Self-Assessment Manikin (SAM) scale in [43,64], UCLA Loneliness Scale (UCLA-LS) in [65], PHQ9 Depression Screener (PHQ9-DS) in [65], Mental Health Continuum Short Form (MHC-SF) in [65], Positive and Negative Affect Schedule (PANAS) in [66], Big Five Inventory (BFI) in [66], and Geriatric Depression Scale (GDS) in [66]. The SAM is a pictorial scale used by two studies, i.e., [43,64], to measure the pleasure, arousal, and dominance of participants. The UCLA-LS, PHQ9-DS, and GDS are standardized questionnaires to measure the loneness, social isolation, and depression of participants. The MHC-SF, PANAS, and BFI assess the overall affects and emotions of participants.

Three standardized questionnaires were used for measuring the usability of SASs. They are the System Usability Scale (SUS) in [17,54,58], Quebec User Evaluation and Satisfaction with Assistive Technology (QUEST) in [46,54,87], and Canadian Occupational Performance Measure tool (COPM) in [50].

(II) Self-developed questionnaires

Three self-developed questionnaires in [18,69,87] were used for measuring the participants’ social interactions and emotional states due to using SASs. For example, in [69], the researchers developed a questionnaire to measure the qualities of life of participants such as psychological aspect and social environment. Ten self-developed questionnaires in [18,28,29,34,48,50,60,77,83,85], measured usability-related aspects of SASs. Usability in these questionnaires consists of several dimensions, such as ease of use in [34,48], learnability [29], user experience in [83,85], and user satisfaction in [60]. 

#### 3.3.3. Observational Data

In total, twenty-four studies collected observational data. A majority of these studies were targeted at users in special age groups. Twelve studies aimed to assist older adults, i.e., [17,59,61,62,67,70,71,72,73,74,76,82]. Seven studies focused on the social problems of children with ASD, i.e., [20,35,38,39,40,41,42]. Five studies were conducted for blind people, i.e., [19,78,85] and people with physical disability, i.e., [52,53].

Generally, the two methods for collecting observational data are taking notes and video recording. Ten studies observed the participants by taking notes, i.e., [17,35,38,40,61,62,70,71,74,76]. In addition to the common form of handwritten texts, checklists and photographs were also adopted in [40,62,74]. The collected data mainly included the participants’ interactions with technologies, social behaviors, and their performances. Given the dynamic nature of behavioral data, notes were often taken by multiple researchers to ensure the integrity and accuracy of the collected data (in [35,38,62,70,71]). Two studies on dementia people specially mentioned that the presence of observers might stress the participants. Therefore, the observers needed to join the participants’ activities or stay out of their view (i.e., in [71,74]). Nine studies used video cameras to collect observational data, i.e., [19,39,41,42,52,72,73,74,85]. They provided more detailed information such as the participants’ verbal data and even facial expressions (in [52,72,73,74]). 

Six studies observed their participants without mentioning their specific collection methods, i.e., [20,53,59,67,78,82]. The observational data are primarily analyzed with qualitative methods, and they are often analyzed with interview data (as in [38,39,40]). Video coding is a common way to analyze videotapes. The coding schemes can be developed by the researchers (as in [39,74]) or adapted from established protocols (as in [52,62,73]). A three-tiered method was utilized in [52] to strengthen the validity of the research in a small sample and heterogeneous populations. In [72], the researchers recorded the participants’ interaction and the computer screen. The videotapes were transcribed separately and analyzed with descriptive methods to investigate the impact of the system on the participants. A thematic analysis was often used to analyze the collected notes to identify emerged categories through open coding (as in [38,40]) or researchers’ discussions based on their research questions (as in [35,38,76]). Other techniques can also be used to facilitate the analysis procedure. For example, a constant comparative analysis was used in [76]. Affinity analysis was adopted in [38,40] to uncover emerged themes. In addition to qualitative methods, quantitative methods can also be applied mainly to count usability issues as in [70]. In [19], a quantitative analysis was used to investigate whether the participants initiated a conversation. Additionally, we found 13 studies did not clearly describe their analytic methods, i.e., [20,41,42,49,53,59,61,67,70,71,78,82,85].

#### 3.3.4. System Log

A total of 11 studies collected data via system logs, targeted people with visual impairments (as in [81,83,85,86,87]), older adults (as in [58,60,75,76]), children with ASD as in [35], and people with SSPI as in [55]. There are two types of system logs; the majority are regarding behavioral logs for social interactions (as in [35,55,58,75,76,81,83,85,86]), and the type is about the usage of the system (as in [60,87]). For example, among behavioral logs for social interactions, three studies logged posted messages as in [58,76] and photos as in [83]. In [58], the researchers performed a qualitative analysis for the content of the posted messages from older adults. Similarly, as reported in [76], the researchers collected sent and received messages from older adults to evaluate how SASs impacted family relationships. Different from [58], the researchers did not analyze the content of the messages due to the requirements of ethical documents. Instead, they analyzed the features of the messages, such as the time of day that messages were sent or received. In [83], the researchers investigated enhancing visually impaired users’ experiences with photos on social networking sites (SNSs). To do so, they extracted behavioral logs of photo engagement actions from target users, such as “liking” or “commenting” on photos. Additionally, in [35], the researchers provided a VR avatar system to regulate social distance for children with ASD. They logged multiple behavioral data, such as the distance from the avatar, volume, and talking duration of participants. Such logs enabled statistical analysis for comparisons to be made between conditions. Other behavioral logs for social interactions included voice recordings, in [55], videos of the request head movements, in [81], and videos of the recognized behavioral expressions, in [85]. In [60,87], researchers analyzed the usage of the system via system logs, such as time, frequency and duration that the system was used. 

#### 3.3.5. User Task Performance 

Ten studies collected data through user task performance. These studies targeted people with visual impairments (as in [28,30,31,32,77,79]), older adults (as in [17,70]), children with ASD (as in [36]), and people with social communication disorders (SCD) (as in [56]). Two types of user task performance were identified as follows:

(I) User task performance for identification of social signals

Eight out of 10 studies used user task performance to identify social signals, such as facial expressions and emotions (in [28,30,31,32,36,56]), head gestures (in [77]), as well as the social distance (in [79]). For example, in [30], the researchers aimed to deliver an interaction of a partner’s facial movements to blind people. In the preliminary study, the participants were asked to select a face image according to vibrotactile cues. Similarly, in [31], the participants identified a given emotion according to the location of the tactile feedback from a belt device. For another example, in [56], the researchers used an affective avatar to engage people with SCD. They tested the participants’ identification accuracies of six avatars’ emotions. The other two studies involved head gesture estimation, in [77], and interpersonal distance recognition, in [79]. In [77], the participants were asked to listen to sonification to estimate head-gestural features. In [79], the researchers tested participants’ recognition accuracies of tactile rhythms to convey the social cue of interpersonal distance for blind people. 

(II) User task performance for evaluating usability

Two out of 10 studies evaluated usability, i.e., [17,70]. In [17], the researchers defined three metrics of the task performance: (a) the task success, (b) the achieved milestone, and (c) the level of assistance, such as how many hints were used for completing a task. In [70], to investigate the effectiveness of SASs, the researchers assigned two tasks to the participants, tracked their number of errors, and time spent completing tasks. 

#### 3.3.6. Functional Validation

Six studies gathered data for functional validation, and targeted people with visual impairments (in [32,34,80,88]), physical disability (in [51]), as well as people with low vision, Alzheimer’s disease, and ASD (in [57]). These studies tested recognition rates of the system, and focused on identifying social signals, such as face recognition (in [34,88]), facial expressions recognition (in [57]), head-nodding recognition (in [80]), and pose detection (in [32,51]). In [88], the researchers used an ESA device to initiate conversation at over two meters and tested the face recognition rate of a friend. Similarly, in [34], the researchers developed a real-time multi-modal system to help blind people access nonverbal cues and tested the face recognition accuracy of interaction partners. One study mentioned that a vision system could detect head-nodding and conveyed this social cue to a blind person via a haptic belt (in [80]). Two studies tested pose detection (in [32,51]). In [32], the researchers provided a social interaction assistant to reduce stereotypic body mannerisms of blind people, which are known to impede social interactions. From motion sensors, researchers examined the detection rate of body rocking. Similarly, in [51], the researchers assessed the pose detection rate for controlling a smart wheelchair system to keep a suitable conversation distance for social following. 

#### 3.3.7. Physiological Data

Four studies measured physiological data of the participants, i.e., [19,33,36,37]. Among them, two studies, i.e., [36,37], measured physiological data of children with ASD. In [36], the researchers developed a VR-based social interaction platform for ASD intervention. The platform collected physiological data of the participants, including eye gaze, EEG signals, and diverse types of peripheral psychophysiological signals, to know their emotional processing and engagement. In [37], the researchers presented a smart waistband to help children with ASD to improve social interactions. One of this band’s functions was to measure their stress level when interacting with others. Galvanic skin response, heart rate, and skin temperature were used to measure the stress level. In a blind-sighted conversation scenario (in [19,33]), gaze data were used to measure the engagement of a sighted interaction partner. 

## 4. Discussion

### 4.1. Insights for Target Users

In this review, we found that the analyzed studies primarily targeted older adults in general, people with visual impairments, and children with ASD. A total of 21 studies developed SASs for older adults. Most of the studies (14 out of 21) targeted older adults in general. The WHO adopts a broad view of “health,” namely “active aging”, considering not only health indicators but also psychological and social aspects [95]. SASs provide a feasible way to enhance the social interactions of older adults in society. Most studies tend to recruit older adults who can accept and use new technologies (as in [69,96]). Such older adults are proficient in using design and expressing their attitudes and feelings. Still, it might cause overlooking the reactions of those in lower levels of acceptance and capability. Seven studies (out of 21) targeted older adults with cognitive impairments. As compared with older adults, they suffered seriously impaired social cognition and changes in their perception and processing of emotions. It is challenging to investigate this type of older adults and find their needs for social interactions. In the analyzed studies, most intervention systems establish convenient connections between older adults with cognitive impairments and their stakeholders, including doctors, family members, and caregivers. Few studies were found that developed a SAS to enhance cognitive ability directly and general communication skills of older adults with cognitive impairments, similar to a social rehabilitation tool for children with ASD. Additionally, it would be meaningful to develop a SAS to help older adults with cognitive impairments, to extend their everyday social circle, and to establish connections beyond stakeholders. We identify these promising research areas as future work.

Twenty studies targeted people with visual impairments. The majority of SASs aim to help blind people perceive social signals, because, during nonverbal communication, most social signals are exchanged through visual cues, such as eye gaze, facial expressions, head pose, and gestures; however, due to a loss of vision, blind people cannot perceive such visual cues, which might cause them to feel socially isolated, especially with sighted people in face-to-face communication. 

Nine studies targeted children with ASD, which indicates an early intervention. A young child’s brain is still forming, which means it is more plastic or changeable than at older ages [97]. Interventions of SASs for children with ASD will be more effective during this stage. 

### 4.2. Insights for Data Gathering

Interviews (N = 31 studies), questionnaires (N = 26 studies), and observations (N = 24 studies) are three major ways of gathering data for evaluating SASs. 

**Interviews are the most often data gathering approach among the reviewed papers**. Interview data mainly aim to address research objectives that are relatively qualitative, open-ended, or exploratory. The types of insights that can be generated from interview data usually concern (a) interviewees’ subjective perspectives, (b) lived experiences (either regarding their existing life or the evaluated assistive systems), and (c) envisaged future scenarios. In many analyzed studies, interview data served as an essential source in triangulating or complementing other types of data (e.g., questionnaires or observational data) to help establish contextualized, specified, or deepened understandings about the experiential aspects of the studied topic. As we found in the analysis, studies that utilized interviews covered a wide range of primary user groups, including older adults (e.g., [63,64]), children with ASD (e.g., [35,40]), and people with visual impairments, hearing loss, physical disability, or communication disorder (e.g., [33,43,78]). One important observation from our analysis is that a considerable proportion of the studies conducted interviews with secondary users, alternative users, or relevant stakeholders to compensate for the perspectives of the primary users. The reason for this is twofold. 

First, some primary users of assistive systems might have difficulties in communication. For instance, older adults with dementia, children with ASD, or people with communication disabilities might face reluctance when engaged in interpersonal conversations. In these cases, interviewing stakeholders or domain experts might bring supplementary understandings about the primary users’ personal preferences or general professional knowledge about the target group. 

Second, in many cases, to design assistive systems should go beyond simply supporting the practical tasks of the primary users. Instead, it also concerns understanding and fulfilling users’ psychological needs in the specific socio-cultural context. In such cases, the perspectives of “others” (e.g., people who are in the same social context as the primary users) should also be studied to better understand the experiences and implications of assistive systems in social settings (e.g., [43]). For example, how an assistive technology would be perceived by others in the context, whether it would create unpreferable social perception for the primary user or inconvenience for others. The above two reasons suggest extra considerations for researchers in conducting interviews in the domain of assistive systems. We also hope to indicate the necessity for future related research to strengthen how interviews and qualitative data analyses are conducted. In our analysis, we found that many studies did not seem to offer detailed information about the script, questions, and operational process of the conducted interviews, which may have weakened the validity of the methodology. In addition, most of the studies provided somewhat limited information about the data analysis process. Many studies simply named a qualitative analysis method without further explaining (a) why the method was opted for, (b) what steps were taken in the actual execution, and (c) how the validity and credibility of the analyses have been guaranteed. Given the abovementioned importance of interview data, we argue that reporting on the methods of conducting interviews and qualitative data analysis should be treated with sufficient rigor, formality, and scrutiny.

**Questionnaires were mostly used for investigating behaviors of older adults (N = 11) and blind people (N = 10).** Standardized questionnaires (N = 18) were more often used than self-developed questionnaires (N = 3) to measure participants’ social interactions and emotional states. Among 11 studies of older adults, most of them (8 studies) used standardized questionnaires to measure their social interactions (in [17,61,62,63]) and emotional states (in [64,65,66]), as well as system usability (in [58]). There were only three studies that used self-developed questionnaires to measure older adults’ social interactions and emotional states, in [18,69], as well as system usability, in [60]. Different from studies of older adults, six out of 10 studies of blind people adopted self-developed questionnaires and focused on investigating system usability (in [28,29,34,77,83,85]). Another three studies (in [19,33,80]) used standardized questionnaires to measure blind participants’ perceptions of social interaction. None of the studies regarding children with ASD used questionnaires since this user group has communicative disabilities, and some children cannot do self-reporting [98]. Questionnaires are not feasible for the researchers to collect behavioral data of children with ASD. Instead, their parents are often in the best placed to report their children’s interests and opinions [98] by using questionnaires and observations. 

Most standardized questionnaires for measuring social interactions (16 out of 18) rely on written text, and only two are pictorial scales. For example, in [64], the researchers used the Inclusion of Other in the Self (IOS) scale and the Self-Assessment Manikin (SAM) scale to test social connectedness between older adults and their caregivers. Older adults could indicate or rate the figure in the pictorial scale that best represented their current emotional state [99]. Thus, the pictural scale enables older adults to report their feelings intuitively and efficiently. In addition, as compared with written text, graphic elements are more friendly for people who cannot correctly read written text, such as children or older adults with age-related lower levels of understanding text questions [100].

**Observational data were mainly collected in the studies for particular age groups: older adults (12 out of 24 studies) and children with ASD (7 out of 24 studies).** Since social problems encountered by target users could present challenges for researchers to collect valid data through interviews and questionnaires, collecting observational data has proven to be an effective way for system evaluation and research validation. The explicit explanations about collecting and analyzing observational data could inform later research in this field, but we found many studies often overlooked them. Generally, video recording contained more detailed and complete data than notetaking that required fewer human resources. Notetaking can be conducted if video recording is not feasible, and it is more suitable for studies with explicit assumptions of users’ behaviors. As [40,74] did, checklists or observational schemes can be made beforehand to reduce the workload of researchers and to improve the efficiency and accuracy of data gathering. The schemes can also be used to facilitate the analysis process. Furthermore, we found most studies made their schemes, and there seems to be a need for standard frameworks to guide researchers to develop observational schemes for evaluating SASs.

**Apart from these three major data gathering methods, only a few studies measured physiological data in system evaluations.** Two studies were focused on children with ASD, i.e., [36,37], and one study measured gaze data of a blind person’s sighted conversation partner. It seemed to be not very common to use physiological data for measuring SASs. Social interactions involve two or more people exchanging ideas and sharing emotions. It is a complicated procedure, which is influenced by many factors. During an evaluation, physiological data might not entirely reflect the actual mental processes and behaviors of participants. Sometimes it might cause misunderstandings of subtle cues of emotions. In addition, measuring specific types of physiological data seems to be not feasible for people with disabilities. For example, many studies measured the conversational engagement of sighted people through their eye gaze data (such as in [101,102]). However, it is not feasible for measuring gaze data of blind people. Although some limitations might exist for physiological data measurements, there have still been several attempts. For example, certain target users are not able to self-report and complete questionnaires, such as children with ASD and older adults with dementia. In [36], the researchers collected physiological data of children with ASD to understand their engagement and emotions, including eye gaze, EEG signals, and different kinds of peripheral psychophysiological signals. Another example is a study by [103], in which the researchers collected physiological and behavioral data of older adults with dementia to establish a model of engagement.

## 5. Conclusions

In this article, we present a state-of-the-art overview of the data gathering methods for SASs. For this systematic review, we analyzed a total of 65 papers, searched from six databases mentioned above. We found that the analyzed studies primarily targeted older adults in general, people with visual impairments, and children with ASD. While this pattern implies the substantial needs for SASs from these three user groups, it may also indicate that the other user groups might be currently underrepresented (e.g., people with a speech disorder and adults with ASD). We believe that it is also meaningful for future research to further compare the proportions of different types of SASs with the statistical distribution of people with disabilities. This comparison could help identify specific user groups that may have been severely under supported. Our research yielded many implications on SASs for specific user groups. For instance, we recommend that SASs for older adults with cognitive impairments should focus on extending the limited everyday social circle of these users, in addition to helping them communicate with their caregivers.

We summarized seven types of data gathering methods for evaluating SASs (i.e., interview data, questionnaires, observational data, user task performance, system log, functional validation, and physiological data). Interviews, questionnaires, and observations were three significant methods of gathering data for evaluating SASs. While these three methods are also frequently used in human-computer interaction studies in general, studies on SASs can face more particular challenges in data gathering due to the communication barriers with their target users. Hence, an open question that remains to be explored is how future researchers could better cope with these barriers, and make participation in this type of study even more inclusive and accessible for target users. More specifically, a considerable proportion of the interview studies involved secondary users, alternative users, or relevant stakeholders in compensating the perspectives of the primary users. This is done because some primary users (e.g., children with ASD or people with communication disabilities) might experience difficulties in interpersonal conversations. Questionnaires were mainly used for older adults and blind people to measure SASs from two aspects: (a) users’ social interactions and emotional states, as well as (b) system usability. Pictural questionnaires (e.g., IOS and SAM) enabled participants with lower levels of understanding text questions to report their perceptions intuitively and efficiently. Observational studies were mainly for particular age groups: older adults and children with ASD. Checklists or observational schemes were proven to be helpful to improve the efficiency and accuracy of data gathering. There is a need to provide standard frameworks for HCI researchers to develop observational schemes for measuring SASs.

Additionally, we found that physiological data were seldom used in system evaluation due to limitations; however, it is still available for certain target users, such as older adults with dementia or children with ASD, who are not able to self-report [104]. Therefore, it is foreseeable that physiological data will be more and more critical for evaluating SASs in future research studies.

## Figures and Tables

**Table 1:** Classification of target users.

Target Population (N)			References
Older adults (21)	Older adults in general (14)		[17,58,59,60,61,62,63,64,65,66,67,68,69,70]
	Cognitive impairments (7)	Dementia	[18,71,72,73,74,75]
		Mild cognitive impairment	[76]
People with disabilities (44)	Visual impairments (20)		[19,28,29,30,31,32,33,34,77,78,79,80,81,82,83,84,85,86,87,88]
	Autism spectrum disorder (ASD) (9)	Children with ASD	[20,35,36,37,38,39,40,41,42]
	Listening and speech impairments (7)	People with deafness	[43,44,45,46,47]
		Deaf and dumb people	[48]
		People with speech or language impairment	[49]
	Mobility and physical impairments (5)	People with physical disabilities	[50,51,52]
		People with motion disability	[53]
		People living with spinal cord injury	[54]
	Others (3)	People with severe speech and physical impairments (SSPI)	[55]
		People with social communication disorders (SCD)	[56]
		People with low vision, Alzheimer’s disease, and ASD	[57]

**Table 2:** Types of data gathering (“N” stands for the number of studies).

Data Gathering	References
Interview data (N = 31)	[18,33,34,35,38,39,40,41,43,44,45,53,54,56,59,60,61,62,63,64,68,70,71,72,76,78,80,82,83,84,87]
Questionnaire (N = 26)	[17,18,19,28,29,33,34,43,46,48,50,54,58,60,61,62,63,64,65,66,69,77,80,83,85,87,89]
Observational data (N = 24)	[17,19,20,35,38,39,40,41,42,52,53,59,61,62,67,70,71,72,73,74,76,78,82,85]
System log (N = 11)	[35,55,58,60,75,76,81,83,85,86,87]
User task performance (N = 10)	[17,28,30,31,32,36,56,70,77,79]
Functional validation (N = 6)	[32,34,51,57,80,88]
Physiological data (N = 4)	[19,33,36,37]

**Table 3:** Questionnaires for measuring SASs (“N” stands for the number of questionnaires).

Questionnaire	Measures	References of Studies
Standardized questionnaire (N = 21)	Social interaction (N = 11)	IOS [19,33,62,64], ABC [63], UES [17], LSNS [65], 2DSIS [80], NMSPI [19], PIADS [54], Life-H [46], FACS-A [46], IAS [61], IMI [33]
	Emotional state (N = 7)	SAM [43,64], UCLA-LS [65], PHQ9-DS [65], MHC-SF [65], PANAS [66], BFI [66], GDS [66]
	Usability (N = 3)	SUS [17,54,58], QUEST ([46,54,87]), COPM [50]
Self-developed questionnaire (N = 13)	Social interaction and emotional state (N = 3)	[18,69,87]
	Usability (N = 10)	[18,28,29,34,48,50,60,77,83,85]

## Data Availability

Not applicable.

## Appendix A

**Table A1:** Summary of studies and features.

No.	References	Research Purpose	Target Users	Methods of Data Gathering
1	Hine and Arnott, 2002, [49]	Design a communication service for people with speech or language impairment to enhance their social interactions when living independently.	People with speech or language impairment (non-speaking people)	Informal observation (usability and usefulness)
2	Miller et al., 2007, [45]	Design a semitransparent video interface to follow discourse at meetings in both collocated and remote settings.	People with deafness	Interview: feedback from interviews after demo sessions
3	Vincent et al., 2007, [46]	Use new assistive technology to evaluate the social participation of people with deafness.	People with deafness	Questionnaires (i.e., social participation, functional communication in activities of daily living, user satisfaction)
4	Nguyen et al., 2008, [50]	Design an interface for people who have physical disabilities and use communication systems by speech-generating devices to make calls and send messages.	People with physical disabilities	Questionnaires: (1) Canadian Occupational Performance Measure tool (COPM8); (2) Equipment Usability Questionnaire.
5	Dadlani et al., 2010, [63]	Use an electronic picture frame to monitor older adults at home with unobtrusive sensors, and collect data about their health, to provide social connectedness to caregivers.	Older adults	1. Interviews 2. Questionnaires: (1) Awareness, connectedness, thinking of each other, usefulness, esthetics, novelty, and fun (2) Privacy, obligations, expectations, and distraction
6	Hirano et al., 2010, [40]	Design an interactive and collaborative visual scheduling system to help children with ASD to understand, structure, and predict activities in their daily lives.	Children with ASD	1. Observations of classroom activities (field notes, photographs and videos) 2. Weekly interviews
7	Krishna et al., 2010, [32]	Develop an assistive technology for blind people, providing them with body rocking feedback and helping them access the facial expressions of their interaction partners.	Blind people	1. Functional validation, detection rate of body rocking 2. User task performance, recognize facial expressions by vibrotactile pattern
8	McDaniel et al., 2010, [79]	Design novel tactile rhythms to convey the social cue of interpersonal distance (proxemics) for blind people.	Blind people	User task performance: users’ recognition accuracies of tactile rhythms
9	Brok and Barakova, 2010, [42]	Design a tangible multiagent system of interactive blocks to establish cooperative play between children with ASD and their caregivers.	Children with ASD	Observation (the participants’ behaviors and interactions with the system)
10	Astell et al., 2010, [74]	Develop a multimedia touch screen system to test whether it can meet the needs of both people with dementia and caregivers to engage in mutually satisfying interactions.	Older adults with dementia	Observation (verbal and nonverbal behaviors)
11	Shim et al., 2010, [61]	An online social gaming environment designed for older adults, to support social interaction through text and voice-based communications.	Older adults	1. Questionnaires (relationship strength between partners) 2. Observation (participants’ behaviors) 3. Interviews (user experience, effectiveness of the system, relationships with social partners)
12	Black et al., 2011, [55]	Design a personal narrative system for children with SSPI.	Children with SSPI	System log (voice recordings from home and school)
13	Gilfeather-Crowley et al., 2011, [88]	Design an enhanced social awareness (ESA) network to assist blind people to identify friends and initiate social contact when their friends are approaching them.	Blind people	Functional validation (recognition error)
14	Escobedo et al., 2012, [39]	Develop a mobile assistive application for children with ASD to extend the social curriculum training and support real-life social situations.	Children with ASD	1. Interviews 2. Observation: (1) Feld notes and video transcripts (2) Video analysis (interactions, social missteps, and topic of conversations)
15	Fuchsberger et al., 2012, [17]	Requirement analysis of an online platform to enhance the intergenerational interactions between geographically distant grandparents and grandchildren.	Older adults	1. Questionnaires: (1) System Usability Scale (2) Self-developed items regarding their engagement 2. Observation (the types of usability issues based on think-aloud protocol and observer protocol) 3. User task performance: (1) Task success (2) Predefined steps for fulfilling the tasks (3) Level of assistance
16	Hermann et al., 2012, [77]	Design a wearable device to represent head movements as sound, aiming at assisting blind people to perceive head gestures.	Blind people	1. User task performance: (1) Accuracy (associate sound with head gestures) (2) Differences between sonification types 2. Questionnaires (the participants’ preferences and expected performances of sonification).
17	Wu and Koon, 2012, [59]	Analyze the computer customization service that can simplify the communication between older adults and caregivers through the tangible and virtual interface in social media.	Older adults	1. Interviews (system usability, behavior, and perception of older adults on using computer) 2. Observation (daily activities of older adults) 3. Interviews (user needs and user feedback)
18	Hourcade et al., 2012, [41]	Design applications that run on multitouch tablets to promote social skills for children with ASD, enabling them to better collaborate, and understand emotions.	Children with ASD	1. Interviews (user feedback) 2. Observation (general user activities)
19	Garattini et al., 2012, [60]	Design and evaluate a communication system prototype to increase interaction in older adults suffering from social isolation and loneliness.	Older adults	1. System log (usage of the system) 2. Interviews (Entry: health, social routines, social routines, changes to social network and experience with technology; Exit: user experience and perceived impact on social connectedness) 3. Interviews (user experiences, further improvement) 4. Questionnaire (user satisfaction and experience)
20	Magee and Betke, 2013, [53]	Design assistive technology to automatically generate a message on the social network to help people with (motion) disabilities to communicate with family and caregivers, to combat loneliness and isolation.	People with (motion) disability	1. Observation 2. Interview (related mainly to usability)
21	Nijhof et al., 2013, [71]	Explore the behavior outcome of a designed technology supporting leisure activity (a social game) of people with dementia, in comparison with a game without technology support.	People with dementia	1. Observation (structured observation using Oshkosh Social Behavior Coding Scale) 2. Interviews with activity facilitators
22	Anam et al., 2014, [29]	Use assistive technology to enable blind people to perceive social signals during a dyadic conversation.	Blind people	Questionnaires: (1) Correctness (2) Learnability (3) Informativeness (4) Usability (5) Portability (6) User satisfaction
23	Bala et al., 2014, [30]	Use a haptic interface to deliver the facial expressions of an interaction partner to blind people.	Blind people	User task performance (select a face image according to vibrotactile cues, to test the recognition rates)
24	Purves et al., 2014, [72]	Develop a touchscreen-based interface to facilitate the communication between people with dementia and their caregivers.	People with dementia	1. Interviews (user experience) 2. Observation (audio transcription and videos on social interaction)
25	Terven et al., 2014, [81]	Present a method of robustly recognizing six head gestures for blind people.	Blind people	1. Observation (head movements) 2. System log (the requested head movement)
26	Louanne, E. and Boyd et al., 2015, [38]	Use collaborative gaming to facilitate social relationships (i.e., membership, partnership, and friendship) in children with ASD.	Children with ASD	1. Observation (field notes) 2. Interviews (interview transcripts)
27	Nazzi et al., 2015, [67]	Design the augmented everyday artifacts (the shopping bag) to make activities of older adults more socially visible to their community, to enhance their face-to-face social interaction.	Older adults	Observation (pictures, videos, and notes experiences of older adults gathered from co-design activities)
28	Abdallah et al., 2016, [48]	Provide an application to transfer text or voice information of hearing people into sign language, aiming at communicating with deaf and dumb people simply and creatively.	Deaf-and-dumb people	Questionnaires about the application: (1) Easy to use (2) Clear instructions (3) Helpful (4) Short response time (5) Willing to use on daily basis
29	Bekele et al., 2016, [36]	Design a multimodal VR-based social interaction platform for ASD intervention, asking children with ASD to recognize the emotions of the virtual characters.	Children with ASD	1. Physiological data: (1) EEG (2) Gaze data (3) Heart rate (HR) (4) Skin temperature (SKT) (5) Respiration rate (RSPR) (6) Galvanic skin conductance rate (SCR) (7) Skin conductance level (SCL) 2. User task performance (the participant was asked to recognize an emotion of the virtual character)
30	Buimer et al., 2016, [31]	Provide vibrotactile feedback through a haptic belt to enhance social interactions for blind people.	Blind people	User task performance (i.e., identify a given emotional according to the location of tactile feedback)
31	Kim et al., 2016, [78]	Design an audio-augmented badminton game to help blind people enjoy physical activities and social interaction with sighted people.	Blind people	1. Interviews (overall experience on the game) 2. Observation (exchanged times of shuttlecock)
32	Sauvé et al., 2016, [69]	Measure the benefits of an online educational game designed for older adults’ quality of life.	Older adults	Questionnaires: self-administered questionnaire (physical state, psychological aspect, social Environment)
33	Tapia et al., 2016, [70]	Explore the effectiveness of a smart TV-based application that promotes social interaction between older adults and their family members through social media.	Older Adults	1. Interviews (system usability) 2. Observation (the participants’ interactions with the system) 3. User task performance (the number of errors, spent time)
34	Wang et al., 2016, [82]	Design a mobile application that not only helps older adults with low vision read better, but also encourages them to interact with family, friends, and society.	Older adults with low vision	1. Observation (interactions with the prototype) 2. Interviews (user feedback)
35	Zhao et al., 2016, [57]	Present an automatic emotion annotation solution on 2.5-D facial data collected from RGB-D cameras.	People with low vision, Alzheimer’s disease, and ASD	Functional validation (the recognition rates)
36	Voss et al., 2016, [20]	Implement a facial expression recognition system based on google glasses, aiming at providing social cues for children with ASD.	Children with ASD	Observation (the effectiveness and usability of the system)
37	Qiu et al., 2016, [33]	Design a Tactile Band to enable the blind person to feel attention (gaze signals) from the sighted person, aiming at enhancing the level of engagement in face-to-face communication.	Blind people	1. Questionnaires (relationship quality, partner closeness) 2. Physiological data (eye gaze) 3. Interviews (participant’s comments and suggestions)
38	Baez et al., 2017, [58]	Design a virtual fitness environment to keep independent-living older adults physically and socially active.	Older adults	1. Questionnaires: (1) Usability (System Usability Scale) (2) Technology acceptance (i.e., anxiety, attractiveness, acceptance, satisfaction, and usefulness) (3) Usefulness by feature 2. System log (qualitative analysis of posted messages, using a coding scheme for measuring the nature of social interactions)
39	Bonnin et al., 2017, [37]	Assist children with ASD to improve social interactions through messages received in a waist smart band.	Children with ASD	1. Observation: an observation instrument included data: (1) Interaction (2) Device (3) Conversation (4) Social errors 2. Physiological data: (1) Galvanic skin response (2) Heart rate (3) Skin temperature
40	Davis et al., 2017, [64]	Use a peripheral activity-based awareness system to capture human activity information, aiming at enhancing context awareness and support social connectedness between older adults and their caregivers.	Older adults	1. A semi-structured interview 2. Interviews: (1) Self-reported moods (the self-assessment manikin (SAM) scale) (2) Social connectedness (IOS scale)
41	Gugenheimer et al., 2017, [43]	Design and evaluate the real-time translation of sign language to promote face-to-face interactions between deaf and hearing people.	People with deafness	1. Interviews (1) Interview video analysis (interaction footage: number of interaction circles) (2) Interview transcripts were coded by two authors 2. Questionnaires for the emotional state (SAM questionnaire): (1) Dominance (2) Arousal (3) Pleasure
42	Meza-de-Luna et al., 2017, [80]	Enhance face-to-face interaction of blind people by conveying the social cue of head-nodding of conversation partners (so that the user could mirror this gesture).	Blind people	1. Questionnaire to evaluate conversation (i.e., the Two-Dimensional Social Interaction Scale) 2. Semi-structured interview (user experiences focusing on how natural the conversation is) 3. Functional validation (head nodding recognition)
43	Papa et al., 2017, [68]	Design a TV-based interface to promote social interaction of less-educated older adults who have difficulty in using computers for socialization.	Older adults with lower education levels and difficulties of using computers	1. Group interviews (opinions about the system) 2. Personal interviews (potential of the system in social inclusion, and QoL improvement)
44	Wu et al., 2017, [83]	Use artificial intelligence to enhance blind people’s experiences with photos on SNSs.	Blind people	1. Interviews (current use of technology and social media, comprehension and general feedback on automatic alt-text) 2. Questionnaires (user experience) 3. System log (photo interactions)
45	Feng et al., 2017, [73]	Evaluate the effectiveness of an Interactive Table Design (ITD) for providing older adults with dementia meaningful engagements.	Older adults with dementia	Observation (emotional, verbal, visual, behavioral, collective engagement, and signs of agitation)
46	Zolyomi et al., 2017, [84]	Understand and investigate the social and emotional impacts associated with the adoption of low-vision assistive technology.	Blind people	Interviews (recall critical incidents, necessary visual qualifications for using eSight, customers’ concerns about adoption, and daily use)
47	Rahman et al., 2017, [85]	Design a smartphone based system to assist blind people to perceive nonverbal signals in natural dyadic conversations.	Blind people	1. Questionnaires (user satisfaction, usability) 2. System log (predicted behavioral expressions) 3. Observation (user behaviors)
48	Sarfraz et al., 2017, [34]	A real-time multi-modal system that provides nonverbal cues (e.g., eye contact, number of people, their names and positions) via audio and haptic interfaces.	Blind people	1. Questionnaire (the usability of interfaces, functions, ease of use, and intuitiveness) 2. Interviews (system’s face identification performance, user acceptance) 3. Functional validation (face recognition accuracy and head orientation accuracy)
49	Louanne, E. and Boyd et al., 2018, [35]	Provide the VR intervention to regulate social distance for children with ASD in social interactions.	Children with ASD	1. System log (user’s distance from the avatar; volume; duration of talking) 2. Interview (interview transcripts) 3. Observation (took detailed paper-pencil field notes)
50	Johnson et al., 2018, [56]	Design and evaluate an affective avatar to engage the user in social interactions to assist in communication therapies.	People with SCD (Social Communication Disorders)	1. User task performance (identification accuracy of six avatar emotions) 2. Interviews (ask user’s opinions whether the emotion of the avatar is logical or not)
51	Lin et al., 2018, [66]	Design a VR system for older adults living in communities, aiming at enhancing their emotional and social well-being.	Older adults	Questionnaires: (1) Health and wellbeing (2) Affects and emotions (e.g., Positive and Negative Affect Schedule (PANAS), big five inventory, Geriatric Depression Scale (GDS)) (3) System user experiences
52	McDaniel et al., 2018, [28]	Design haptic display using vibrotactile representation to convey facial action units (e.g., lip corners pulled up, cheeks raised), to help blind people perceive social signals.	Blind people	1. User task performance (recognition accuracies of participants about the designed vibrotactile representation) 2. Questionnaire (self-report Likert scale)
53	Pingali et al., 2018, [51]	Design an autonomous wheelchair that can perform side-by-side following to reduce the mental load for people with physical disabilities in simultaneously navigating the wheelchair and conversing with people.	People with physical disabilities	Functional validation (sensor sampling rates, wireless communication distance, etc.)
54	Yurkewich et al., 2018, [76]	Evaluate how a tablet-based communication technology designed for older adults was used and impact family relationships.	Older adults with cognitive impairment	1. Interviews (participants’ past experiences and present life, patterns of use, the facilitators and barriers to use, user experience) 2. Ethnography (field notes recording training strategies, activities practiced) 3. Observation (use scenarios) 4. System log (sent and received messages)
55	Fleury et al., 2019, [87]	Design and evaluate a fabric-based speech-generating device (SGD) for nonverbal pediatric participants with vision impairment.	The nonverbal pediatric participant with vision impairment	1. Questionnaires completed by caregivers: (1) User acceptance (QUEST 2.0) (2) The participant’s feelings regarding her communication devices 2. System log (daily usage statistics)
56	Isaacson et al., 2019, [65]	Design and evaluate a communication system, aiming at promoting social connectivity and providing entrainment content for older adults.	Older adults	Questionnaires: (1) Loneliness (UCLA Loneliness Scale) (2) Depression (PHQ9 Depression Screener) (3) Emotional wellbeing (Mental Health Continuum Short Form (MHC-SF)) (4) Social engagement (Lubben Social Network Scale)
57	Marti and Recupero, 2019, [44]	Design smart jewels to support people with hearing loss beyond functional needs: instead of supporting hearing, the system aims to support emotional and socio-cultural needs.	People with deafness or hearing loss	In-depth interview (lived experiences on aesthetics, self-expression, and identity)
58	McCarron et al., 2019, [18]	Support people with memory loss (dementia) to remember people (names and relationships) that they interact in social activities, to increase their social engagement.	People with memory loss	1. Questionnaires (quality of social interactions and quality of life) 2. Semi-structured interviews
59	Tamplin, et al., 2019, [54]	Test the acceptability and feasibility of an online virtual reality platform for therapeutic group singing interventions for people living with spinal cord injury.	People living with spinal cord injury	1. Questionnaires (the overall user experience) 2. Interviews (user experience)
60	Lee et al., 2020, [86]	Design a working prototype for pedestrian detection, to decrease the social tensions of blind people.	Blind people	System log (the pedestrians’ attributes, such as name, gender, head pose, and position)
61	Li et al., 2020, [62]	Design three interactive systems to enhance the social interaction of older adults.	Older adults	1. Questionnaire (IOS) 2. Semi-structured interviews 3. Observation (video recordings)
62	Qiu et al., 2020, [19]	Design Social Glasses system to let a blind person perceive and react “eye gaze” to a sighted conversation partner, to enhance their communication quality.	Blind people	1. Questionnaire (NMSPI, IOS) 2. Observation (video recordings) 3. Physiological data (eye gaze)
63	Theil et al., 2020, [47]	Present a mobile Augmentative and Alternative Communication (AAC) device to facilitate communication of deaf-blind people.	Deaf blind people	N/A
64	Bellini et al., 2021, [75]	Analyze the localization data of AD patients living in assisted care homes to understand their social behaviors, promote their sociability and delay cognitive decline.	AD patients (Alzheimer’s Disease)	System log
65	Hsieh et al., 2021, [52]	Use eye-gaze assistive technology to promote dyadic interaction between children with severe physical disabilities and their communication partners.	Children with physical disabilities and complex communication needs	Observation (video-coding for communicative interaction, observational scheme)

## Appendix B

Published journals and conference papers during 2000–2021 regarding SASs based on classification of the target population.

**Older adults in general** [17] *International Conference on Computers for Handicapped Persons* [58] *International Conference on Collaboration Technologies and Systems* [59] *International Convention on Rehabilitation Engineering and Assistive Technology* [60] *Universal Access in the Information Society* [61] *International Academic Conference on the Future of Game Design and Technology* [62] *Sustainability* [63] *Ai & Society* [64] *IEEE Access* [65] *International Conference on Technologies for Active and Assisted Living* [66] *International Conference on Human Aspects of IT for the Aged Population* [67] *International Conference on Human Aspects of IT for the Aged Population* [68] *Informatics for Health and Social Care* [69] *Communications in Computer and Information Science* [70] *International Conference on Ubiquitous Computing and Ambient Intelligence*
**Older adults with cognitive impairments** [18] *JMIR aging* [71] *Technology and Disability* [72] *American Journal of Alzheimer’s Disease & Other Dementias^®^* [73] *International Conference on Smart Health* [74] *Interacting with Computers* [75] *Sensors* [76] *International Journal of Mobile Human Computer Interaction*
**People with visual impairments** [19] *IEEE Access* [28] *Proceedings of the 2018 Workshop on Multimedia for Accessible Human Computer Interface* [29] *International Joint Conference on Pervasive and Ubiquitous Computing* [30] *2014 IEEE International Symposium on Haptic, Audio and Visual Environments and Games* [31] *International Conference on Human-Computer Interaction* [32] *International Conference on Computers for Handicapped Persons* [33] *International Conference on Universal Access in Human-Computer Interaction* [34] *Informatik-Spektrum* [77] *Proceedings of the 7th Audio Mostly Conference: A Conference on Interaction with Sound* [78] *CHI Conference on Human Factors in Computing Systems* [79] *CHI Conference on Human Factors in Computing Systems* [80] *International Journal of Human-Computer Studies* [81] *Mexican Conference on Pattern Recognition* [82] *CHI Conference on Human Factors in Computing Systems* [83] *ACM Conference on Computer-Supported Cooperative Work* [84] *ACM SIGACCESS Conference on Computers and Accessibility* [85] *Multimedia Tools and Applications* [86] *CHI Conference on Human Factors in Computing Systems* [87] *Disability and Rehabilitation: Assistive Technology* [88] *IEEE International Conference on Systems, Man and Cybernetics*
**Children with ASD** [20] *ACM International Joint Conference on Pervasive and Ubiquitous Computing* [35] *CHI Conference on Human Factors in Computing Systems* [36] *IEEE Virtual Reality Conference* [37] *International Conference of Design, User Experience, and Usability* [38] *ACM Transactions on Accessible Computing* [39] *CHI Conference on Human Factors in Computing Systems* [40] *CHI Conference on Human Factors in Computing Systems* [41] *Personal and ubiquitous computing* [42] *International conference on entertainment computing*
**People with listening and speech impairments** [43] *ACM Conference on Computer-Supported Cooperative Work* [44] *ACM Creativity and Cognition* [45] *ACM Southeast Regional Conference* [46] *Technology and Disability* [47] *International Conference on Mobile and Ubiquitous Multimedia* [48] *Procedia Computer Science* [49] *ACM conference on Assistive technologies*
**People with mobility and physical impairments** [50] *Technology and Disability* [51] *Sensors* [52] *International journal of environmental research and public health* [53] *International Conference on Universal Access in Human-Computer Interaction* [54] *Journal of telemedicine and telecare*
**Others** [55] *ACM conference on Assistive technologies* [56] *Health informatics journal* [57] *IEEE transactions on cybernetics*
